# Malnutrition Among Children Under Five in Djibouti: A Composite Index of Anthropometric Failure Analysis from the 2023 Multisectoral Survey

**DOI:** 10.3390/nu18020306

**Published:** 2026-01-19

**Authors:** Hassan Abdourahman Awaleh, Tony Byamungu, Mohamed Hsairi, Jalila El Ati

**Affiliations:** 1 Department of Health, University of Senghor, Quartier des Universités, Axe Central, Ville Borg El-Arab El-Gedida 5220220, Egypt; hassan.awaleh.ed2022@etu-usenghor.org; 2 National Nutrition Program, Ministry of Health, Djibouti City P.O. Box 296, Djibouti; 3 Unicef, Djibouti City P.O. Box 583, Djibouti; tbyamungu@unicef.org; 4 Faculty of Medicine of Tunis, University of Tunis El Manar, Tunis 1068, Tunisia; mohamed.hsairi@yahoo.fr; 5 SURVEN (Nutrition Surveillance and Epidemiology in Tunisia) Research Laboratory, INNTA (National Institute of Nutrition and Food Technology), 11 Rue Jebel Lakhdar, Bab Saadoun, Tunis 1007, Tunisia; 6 University of Tunis El Manar, Tunis 1068, Tunisia

**Keywords:** child undernutrition, composite index of anthropometric failure (CIAF), malnutrition, children under fine, Djibouti

## Abstract

**Background/Objectives**: Child undernutrition remains a major public health in Djibouti, yet conventional anthropometric indicators may underestimate its true burden by failing to capture overlapping forms of malnutrition. The Composite Index of Anthropometric Failure (CIAF) provides a more comprehensive assessment by identifying children experiencing one or multiple anthropometric deficits. This study aimed to estimate the prevalence and determinants of undernutrition among children under five years of age in Djibouti using the CIAF. **Methods**: This study is a secondary analysis of data from the nationally representative 2023 Multisectoral Survey conducted in Djibouti. A cross-sectional design with a two-stage stratified cluster sampling method was used to collect data on a national random sample (*n* = 2103) of children aged 6–59 months. Standardized anthropometric measurements were used to derive conventional indicators (stunting, wasting, and underweight) and the CIAF. Binary logistic regression analyses were performed to identify factors associated with anthropometric failures, adjusting for child, household, and contextual characteristics. **Results**: Based on conventional indicators, 23.4% of children were stunted, 20.0% were underweight, and 9.9% were wasted. Using the CIAF, 36.9% of children experienced at least one anthropometric failure, including 18.8% with multiple concurrent failures. Boys, children aged 6–47 months, those living in nomadic households, and those residing in specific regions had significantly higher risks of undernutrition. Socioeconomic indicators and household food security were not independently associated with undernutrition after adjustment. **Conclusions**: More than one-third of children under five in Djibouti experience undernutrition when assessed using the CIAF, revealing a substantial hidden burden not captured by conventional indicators alone. Incorporating the CIAF into routine nutrition surveillance could improve identification of vulnerable children and support more targeted, context-specific interventions.

## 1. Introduction

Child undernutrition remains a major public health concern in low- and middle-income countries, despite significant progress in expanding child health services and nutrition interventions [1,2]. It manifests primarily as stunting, wasting, and underweight, conditions strongly associated with increased morbidity, mortality, and long-term cognitive impairment among children under five years of age [3,4]. According to the World Health Organization (WHO), nearly half of all deaths in this age group worldwide are attributable to undernutrition [5].

Globally, stunting affects an estimated 148 million children under five, making it one of the most widespread forms of undernutrition [6]. Sub-Saharan Africa bears the highest burden, where approximately one in three children is affected [6]. In parallel, about 50.5 million children worldwide suffer from wasting, including 17 million with severe forms [7]. Overall, undernutrition affects an estimated 264 million people across Sub-Saharan Africa, representing about one-quarter of the region’s population [8]. The risk of stunting, wasting, and underweight is influenced by multiple and interrelated determinants, including inadequate infant and young child feeding practices, recurrent infections, poor maternal nutrition, poverty, food insecurity, limited access to water, sanitation and hygiene (WASH), weak health systems, and insufficient prenatal and postnatal care [9,10,11,12].

Djibouti, located in the Horn of Africa, exemplifies the persistence of this nutritional challenge. With a population of approximately 1.2 million [13], the country faces severe structural constraints due to its arid climate and limited agricultural production, leading to heavy reliance on food imports. Recurrent food insecurity, high poverty levels, and the added pressures of displacement and urbanization have compounded the risk of child undernutrition [14]. National data indicate that 20.9% of children under five are stunted and 10.1% are wasted, highlighting a substantial and ongoing public health concern. However, available evidence remains fragmented and insufficient to fully understand the socio-economic, environmental, and healthcare-related factors shaping child nutritional outcomes [15].

Djibouti represents a critical gap in the global nutrition literature. Most existing evidence originates from multi-country analyses or regional estimates that obscure local heterogeneity and fail to reflect the country’s unique demographic, socioeconomic, and ecological realities. There is limited knowledge of how household characteristics, infant and young child feeding practices, WASH access, food security, and displacement dynamics interact to influence child growth in this setting. The scarcity of recent, nationally representative analyses hinders the formulation of evidence-based nutrition policies and weakens the country’s ability to monitor progress toward national and global nutrition targets.

Methodologically, conventional anthropometric indicators (stunting, wasting, and underweight) are often examined separately, which may underestimate the true extent of child undernutrition. Many children experience multiple forms of anthropometric failure simultaneously [16,17,18]. To address this limitation, the Composite Index of Anthropometric Failure (CIAF) was developed as an integrated measure that captures overlapping and multiple nutritional deficits. By combining stunting, wasting, and underweight into a single metric, the CIAF provides a more comprehensive estimate of the overall burden of undernutrition and identifies hidden forms of deprivation not detected by individual indicators [17,19,20]. Evidence from several countries across Africa and Asia has demonstrated its utility in revealing the multifaceted nature of child undernutrition and informing more targeted and effective interventions [21,22,23,24].

In this context, the present study aims to estimate the prevalence and determinants of undernutrition among children aged 6–59 months in Djibouti using the Composite Index of Anthropometric Failure. By leveraging data from the 2023 Multisectoral Survey, this study provides the first nationally representative CIAF-based analysis in the country, aiming to fill critical data gaps and generate actionable insights to inform policy and guide nutrition programming.

## 2. Materials and Methods

### 2.1. Study Area

Djibouti, a low-income country ranked 178th out of 204 on the 2022 Human Development Index, is located in the Horn of Africa. It is bordered by Eritrea to the north, Ethiopia to the west and south, and Somalia to the southeast, with a coastline along the Red Sea and the Gulf of Aden. The country covers approximately 23,200 km^2^ and has an estimated population of about 1.2 million, the majority residing in the capital, Djibouti City. Despite rapid urbanization, poverty, unemployment, and limited access to essential services remain widespread, especially in rural and peri-urban areas. The predominantly arid to semi-arid climate, characterized by high temperatures, low rainfall, and recurrent droughts, severely constrains agricultural production. As a result, Djibouti is highly dependent on food imports, contributing to recurrent food insecurity and vulnerability to price fluctuations [13,25,26].

### 2.2. Study Design and Sampling

This study is a secondary analysis of data from the 2023 Multisectoral Survey conducted in Djibouti in March 2023. The survey employed a cross-sectional design and collected data from a nationally representative sample of households to assess determinants of malnutrition among children aged 6–59 months. The survey targeted all households with at least one child within this age range. In households with more than one eligible child, all children aged 6–59 months were included in the survey. Statistical analyses accounted for the clustered survey design, with households treated as the primary sampling unit. Data were collected on child dietary practices, nutritional status, and healthcare access, as well as household socio-economic and environmental conditions. A two-stage stratified cluster sampling design was used to ensure representativeness.

- First stage: The country was divided into enumeration areas (EAs), geographical units averaging 90 households, based on census maps. EAs were randomly selected using probability proportional to population size within each stratum (region or sector), using ENA Software for SMART (Version 2011, 31 July 2012).
- Second stage: Within each selected EA, a fixed number of households was randomly chosen using a systematic random sampling approach.

### 2.3. Data Collection

Data were collected through face-to-face interviews with household respondents using pre-programmed questionnaires on digital tablets. The survey comprised two main components:

- Household component: demographic characteristics, housing and living conditions, access to water, hygiene and sanitation, sources of income and livelihoods, and household food security.
- Child component: breastfeeding and complementary feeding practices, morbidity and healthcare access, and anthropometric measurements.
- Field teams were trained and supervised to ensure consistency and data quality throughout the survey.

### 2.4. Anthropometric Measurements

Anthropometric data were collected using standardized procedures following the WHO Child Growth Assessment Protocol [17,27]. Three conventional indicators were measured: weight-for-age (WAZ), height/length-for-age (HAZ), and weight-for-height/length (WHZ). The Composite Index of Anthropometric Failure (CIAF) was subsequently derived from these indices to assess overall undernutrition. Age in months was calculated based on the birth date reported by the mother or caregiver.

#### 2.4.1. Weight-for-Age

Children’s weights were measured using a SECA 876 digital floor scale (capacity 250 kg; precision 0.01 kg) equipped with a “mother–child” function. For younger children unable to stand unassisted, the mother was first weighed alone and then while holding the child, with the scale automatically computing the child’s net weight. Children were weighed wearing light clothing and no footwear, with all accessories removed. Scales were calibrated daily. Nutritional status was categorized using WHO growth standards: underweight (WAZ < –2 SD) and severely underweight (WAZ < –3 SD) [28,29].

#### 2.4.2. Length/Height-for-Age

Length (for children under two years or unable to stand) was measured in the supine position using a standard length board (precision 0.1 cm). For children aged two years or older, height was measured in the standing position using a calibrated stadiometer. Children were measured without shoes or head coverings, standing or lying straight with heels, buttocks, and shoulders in contact with the board. Nutritional status was classified as stunted (HAZ < –2 SD) or severely stunted (HAZ < –3 SD) [28,29].

#### 2.4.3. Weight-for-Height/Length

The weight-for-height/length index was used to assess acute malnutrition (wasting), which reflects current nutritional status independent of age. The same standardized weighing and measuring procedures were applied. Nutritional status was classified as wasted (WHZ < –2 SD), severely wasted (WHZ < –3 SD), or normal [28,29].

### 2.5. Outcome Variable

The main outcome variable was the CIAF, which captures the overall prevalence of undernutrition by identifying children experiencing single or multiple anthropometric failures. A child was categorized as undernourished if one or more of the following conditions were present: wasting, stunting, or underweight (each defined as z-score < –2 SD from the WHO reference median) [17,27].

The CIAF classifies children into seven mutually exclusive categories (Table 1) based on combinations of anthropometric deficits [28].

### 2.6. Independent Variables

Independent variables encompassed child, household, and contextual characteristics.

- Child-level: gender (boy, girl) and age group (6–23, 24–47, 48–59 months).
- Household-level: area of residence (urban, rural), household type (sedentary, nomadic), and region (Djibouti-City, Balbala, Ali-Sabieh, Dikhil, Tadjourah, Obock, Arta).
- Socioeconomic characteristics: education level of household head (none/primary, secondary, higher), occupation (unemployed/dependent on aid, worker/employee, mid- or senior-level employee, small or large trader). Household economic status was assessed using two complementary indices: a wealth index and an income index. The wealth index was derived using principal component analysis (PCA) of housing characteristics (type of dwelling, persons per room, drinking water, lighting, cooking fuel) and household asset ownership. The first principal component was retained, and households were classified into low, middle, and high wealth groups based on tertiles [31,32,33]. The income index was based on total household income self-reported for the month preceding the survey and similarly categorized into tertiles.
- Household food security: assessed using the Consolidated Approach for Reporting Indicators of Food Security (CARI) and categorized as food secure, marginally food secure, moderately food insecure, or severely food insecure [34].

### 2.7. Statistical Analysis

All analyses were conducted using Stata version 16.0 (StataCorp, College Station, TX, USA). Sampling weights were applied to account for survey design and ensure national representativeness.

The analytical strategy followed three sequential steps. First, descriptive analyses were conducted to estimate the weighted prevalence of individual anthropometric failures (wasting, stunting, underweight) and CIAF categories. Second, bivariate analyses were performed to examine associations between CIAF categories and independent variables using binary logistic regression; variables with a *p*-value < 0.05 were considered for inclusion in the multivariate models. Third, multivariate binary logistic regression analyses were carried out to identify factors independently associated with CIAF categories. Potential confounders were identified a priori based on existing literature and included child’s gender; age in months, categorized as 6–23, 24–47, and 48–59 months; area of residence (urban/rural); household type (nomadic/sedentary); region of residence; education level of the household head (low, middle, high); occupation of the household head (large-scale trader, worker/employee, senior or middle manager, unemployed/dependent on aid, small-scale trader); household wealth index (low, middle, high); household income index (low, middle, high); and household food security status assessed using the CARI index (severely food insecure, moderately food insecure, marginally food secure, food secure). These variables were entered simultaneously into the multivariate models to control for confounding effects. Model adequacy for binary logistic regression analyses assessing associations with wasting, stunting, underweight, and any anthropometric failure was evaluated using the Hosmer–Lemeshow goodness-of-fit test, which indicated satisfactory model fit. Multicollinearity among covariates was assessed using Variance Inflation Factors (VIFs) derived from auxiliary linear regression models including the same independent variables; all VIF values were below the commonly accepted threshold, indicating no evidence of problematic multicollinearity. Results from binary logistic regression analyses were reported as relative risk ratios (RRRs) with 95% confidence intervals (95% CI) and corresponding *p*-values. A two-sided *p*-value < 0.05 was considered statistically significant.

## 3. Results

### 3.1. Subjects

Among the 2103 surveyed children, 36 were excluded due to absence at the time of measurement, missing information, or flagged anthropometric data and a total of 2067 children were included in the analyses.

### 3.2. Prevalence of Malnutrition in Children Under Five Years

#### 3.2.1. Prevalence of Malnutrition According to Conventional Indices and the CIAF Approach

**Figure 1:**
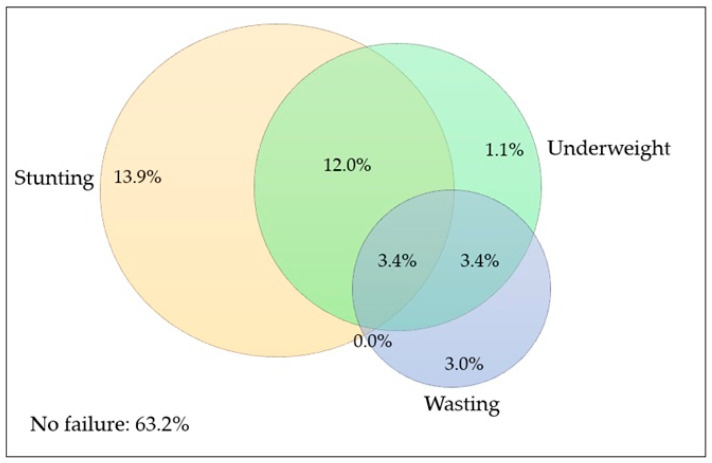
Overlap between wasting, stunting, and underweight among children aged 6–59 months in Djibouti (*n* = 2067).

Based on conventional anthropometric indicators, 29.2% of children were stunted, 19.9% were underweight, and 9.8% were wasted (Table 2). Further disaggregation using the CIAF categories revealed that 13.9% of children were stunted only, 3.0% were wasted only, 1.1% were underweight only, and 12.0% were both stunted and underweight. Multiple concurrent failures were also observed: 3.4% of children were both wasted and underweight, and 3.4% experienced all three forms simultaneously (Table 2, Figure 1).

#### 3.2.2. Gender Differences in Malnutrition

Gender-specific patterns were observed. Boys were significantly more likely than girls to be wasted (11.6% vs. 8.0%, *p* = 0.006) and to experience any form of anthropometric failure (39.3% vs. 34.2%, *p* = 0.02) (Table 2).

Venn diagram illustrating the distribution and overlap of the three conventional anthropometric indicators of undernutrition: wasting (weight-for-height z-score < −2 SD), stunting (height-for-age z-score < −2 SD), and underweight (weight-for-age z-score < −2 SD), based on WHO Child Growth Standards. Values represent weighted prevalence (%) of children experiencing single or multiple anthropometric failures. The diagram highlights the coexistence of undernutrition forms and complements the Composite Index of Anthropometric Failure (CIAF) classification.

### 3.3. Factors Associated with Wasting

#### 3.3.1. Crude Analysis

In the univariate analysis, several sociodemographic factors were significantly associated with wasting among children under five in Djibouti (Table 3). Girls had a significantly lower risk of wasting compared to boys (RRR = 0.7; 95% CI: 0.49–0.88; *p* = 0.006). Age also played an important role: children aged 24–47 months were more likely to be wasted than those aged 48–59 months (RRR = 1.5; 95% CI: 1.00–2.32; *p* = 0.045). A marked difference was observed by household mobility status: children from sedentary households had a significantly lower risk of wasting compared to those from nomadic households (RRR = 0.6; 95% CI: 0.46–0.83; *p* = 0.001). Geographical disparities were also evident: the prevalence of wasting was particularly high in Obock, where children were more than twice as likely to be wasted than those in Dikhil (RRR = 2.1; 95% CI: 1.18–3.86; *p* = 0.012). No statistically significant associations were found between wasting and the education level or occupation of the household head, household wealth, or food security status in the univariate models.

#### 3.3.2. Adjusted Analysis

After adjustment for potential confounders, the associations observed remained robust (Table 3). Girls continued to be associated with a significantly lower risk of wasting (RRR = 0.6; 95% CI: 0.44–0.82; *p* = 0.0013). Children aged 24–47 months remained at higher risk compared to those aged 48–59 months (RRR = 1.5; 95% CI: 1.01–2.38; *p* = 0.044), confirming that this age group represents a critical vulnerability window. The protective effect of sedentary residence persisted strongly after adjustment (RRR = 0.3; 95% CI: 0.20–0.58; *p* < 0.0001), suggesting that nomadic living conditions substantially increase exposure to wasting. Regional disparities were even more pronounced in the multivariate model: children living in Obock (RRR = 3.1; 95% CI: 1.64–5.98; *p* = 0.0005) and Ali-Sabieh (RRR = 2.2; 95% CI: 1.11–4.49; *p* = 0.02) faced significantly higher risks compared to Dikhil. No independent association was observed between wasting and household wealth, income, or food security after adjustment.

### 3.4. Factors Associated with Stunting

#### 3.4.1. Crude Analysis

Univariate analyses identified age, household type, and region as key determinants of stunting among children under five in Djibouti (Table 4). Younger age was strongly associated with higher stunting risk: children aged 6–23 months were significantly more likely to be stunted compared with those aged 48–59 months (RRR = 1.7; 95% CI: 1.27–2.21; *p* < 0.001). Children aged 24–47 months also showed a higher, though borderline, risk (RRR = 1.3; 95% CI: 0.98–1.78; *p* = 0.06). Children from sedentary households were less likely to be stunted compared to those from nomadic households (RRR = 0.6; 95% CI: 0.50–0.73; *p* < 0.001). Marked regional disparities were also observed. The highest prevalence of stunting was recorded in Tadjourah (36.3%) and Ali-Sabieh (34.6%), while the lowest was found in Djibouti City (23.2%). Children in Obock showed a higher risk of stunting than those in Dikhil (RRR = 1.3; 95% CI: 0.89–1.92; *p* = 0.17). Socioeconomic factors, such as household wealth and income, were associated with stunting in the univariate analysis. Children from low-income and low-wealth households had significantly higher odds of being stunted compared to those from wealthier households (RRR = 1.9; 95% CI: 1.48–2.38; *p* < 0.001, and RRR = 1.6; 95% CI: 1.24–2.02; *p* = 0.001, respectively). However, food security status showed no significant association.

#### 3.4.2. Adjusted Analysis

After adjusting for potential confounders, several associations remained significant (Table 4). Children aged 6–23 months (RRR = 1.7; 95% CI: 1.26–2.22; *p* < 0.001) and those aged 24–47 months (RRR = 1.4; 95% CI: 1.00–1.85; *p* = 0.044) were at significantly higher risk of stunting than those aged 48–59 months. This indicates that the period from 6 to 47 months is a critical window for growth faltering in Djibouti. Children from sedentary households remained significantly less likely to be stunted than those from nomadic ones (RRR = 0.7; 95% CI: 0.47–0.93; *p* = 0.017), confirming the protective effect of settled living conditions. Regional disparities persisted after adjustment: children living in Obock had a significantly higher risk of stunting compared to those in Dikhil (RRR = 1.6; 95% CI: 1.03–2.41; *p* = 0.032). In contrast, associations with household wealth, income, and parental education were no longer statistically significant after adjustment, suggesting that other contextual or environmental factors may play a more influential role in child linear growth.

### 3.5. Factors Associated with Underweight

#### 3.5.1. Crude Analysis

In crude models, underweight prevalence did not differ by gender or age (Table 5). Children living in rural areas were significantly more affected than those in urban settings (24.0% vs. 16.1%; RRR = 0.6; 95% CI: 0.48–0.75; *p* < 0.0001). Children from sedentary households had a substantially lower risk than those from nomadic households (RRR = 0.6; 95% CI: 0.46–0.72; *p* < 0.0001). Pronounced regional disparities were observed, with the highest prevalence in Obock (30.1%). Higher educational attainment of the household head and higher household wealth and income were associated with lower risks of underweight, whereas household head occupation and food security status were not significantly associated.

#### 3.5.2. Adjusted Analysis

After adjustment, sedentary household type remained independently protective against underweight (RRR = 0.7; 95% CI: 0.45–0.96; *p* = 0.030). Residence in Djibouti-ville was associated with a reduced risk (RRR = 0.7; 95% CI: 0.46–0.96; *p* = 0.030), while residence in Arta was associated with an increased risk (RRR = 1.7; 95% CI: 1.08–2.66; *p* = 0.023). The elevated risk observed in Obock in crude analyses was reversed after adjustment, with Obock showing a protective association (RRR = 0.4; 95% CI: 0.17–0.75; *p* = 0.007), suggesting strong confounding by socioeconomic and household characteristics. Other socioeconomic variables were no longer independently associated with underweight (Table 5).

### 3.6. Factors Associated with Any Anthropometric Failure

#### 3.6.1. Crude Analysis

In crude models, girls were less likely than boys to experience any anthropometric failure (RRR = 0.8; 95% CI: 0.66–0.95; *p* = 0.015). Children aged 6–23 months and 24–47 months had significantly higher risks compared with those aged 48–59 months. Sedentary household type was associated with a higher risk in crude analysis (RRR = 1.7; 95% CI: 1.41–2.02; *p* < 0.0001), while urban residence was protective. Regional disparities were evident, with lower risk in Tadjourah and higher risk in Obock. Lower household wealth, income, and education were also associated with increased risk (Table 6).

#### 3.6.2. Adjusted Analysis

After adjustment, girls remained protective (RRR = 0.7; 95% CI: 0.61–0.89; *p* = 0.001), and younger age groups (6–23 and 24–47 months) remained at significantly higher risk. Sedentary household type became protective in the adjusted model (RRR = 0.6; 95% CI: 0.45–0.85; *p* = 0.003), indicating confounding in crude analyses. Obock consistently emerged as a high-risk region (RRR = 1.6; 95% CI: 1.08–2.42; *p* = 0.018), whereas other regional associations were no longer significant. Socioeconomic indicators and food security status were not independently associated with anthropometric failure after adjustment (Table 6).

A summary comparison of key factors associated with all malnutrition indicators is presented in Table 7.

## 4. Discussion

This study provides the first nationally representative assessment of child undernutrition in Djibouti using the CIAF, offering a more comprehensive understanding of nutritional vulnerability than conventional anthropometric indicators alone. The findings reveal that more than one-third of children aged 6–59 months (36.9%) experience at least one form of anthropometric failure, highlighting undernutrition as a persistent and substantial public health challenge in Djibouti, despite ongoing nutrition and social protection efforts.

### 4.1. Added Value of the CIAF Approach

The CIAF estimate substantially exceeds the prevalence obtained from individual indicators such as stunting, wasting, or underweight considered separately, confirming that reliance on conventional measures alone underestimates the true burden of child undernutrition. Importantly, the CIAF identified a considerable proportion of children experiencing multiple concurrent failures, particularly the combination of stunting and underweight (12%) and the coexistence of all three failures (3.4%). These children represent a highly vulnerable subgroup with elevated risks of morbidity, mortality, and long-term developmental impairment.

The magnitude of CIAF observed in Djibouti is comparable to that reported in several East and Horn of Africa countries, including Ethiopia and Tanzania (approximately 37–42%), while higher levels have been observed in South Asia contexts such as India and Bangladesh, where CIAF prevalence often exceeds 45% [35,36,37,38,39,40,41,42,43]. These differences likely reflect variations in food system resilience, exposure to environmental shocks, maternal nutrition, and health system capacity. In Djibouti, chronic aridity, dependence on food imports, recurrent drought, and population mobility appear to generate a nutritional burden similar to that seen in other structurally vulnerable settings. Overall, these findings reinforce the relevance of the CIAF as a robust and policy-relevant metric for capturing the multidimensional nature of child undernutrition, particularly in contexts where multiple deficits frequently coexist.

### 4.2. Gender and Age Patterns of Undernutrition

Gender and age differentials in undernutrition observed in this study are broadly consistent with evidence from other low-income settings, with boys and younger children exhibiting higher vulnerability. Importantly, the CIAF adds nuance by demonstrating that these groups are not only more likely to experience isolated forms of undernutrition, but are also disproportionately affected by multiple concurrent anthropometric failures.

The higher CIAF prevalence among boys suggests a cumulative nutritional disadvantage that may not be fully captured by wasting or stunting alone. Similarly, the concentration of CIAF among children aged 6–47 months reinforces the critical importance of early childhood, a period during which overlapping nutritional deficits frequently emerge [30]. This pattern is consistent with global evidence showing that growth faltering accelerates after infancy when children transition to family foods that are often nutritionally inadequate and when exposure to infections increases [44,45,46,47,48]. These findings highlight the need for integrated early-life interventions addressing diet quality, infection prevention, and caregiving practices simultaneously.

### 4.3. Household Mobility and Regional Disparities

Household mobility and geographic location emerged as strong determinants of child undernutrition, with their implications most clearly revealed through the CIAF. Children living in nomadic households experienced substantially higher levels of overall anthropometric failure, reflecting layered vulnerabilities related to limited access to health services, safe water, diversified diets, and social protection [49]. Similarly, Obock consistently emerged as a high-risk region for CIAF, even after adjustment for household-level socioeconomic factors. This persistence suggests that structural and contextual determinants, such as geographic isolation, recurrent drought, and limited service coverage, contribute to simultaneous anthropometric failures. The CIAF thus provides added value by identifying populations where fragmented or single-outcome interventions may be insufficient, underscoring the need for coordinated, multisectoral responses.

### 4.4. Socioeconomic Factors and Food Security

Although socioeconomic variables and food security were associated with undernutrition in crude analyses, their effects were attenuated after adjustment, suggesting indirect pathways of influence. The CIAF offers an integrative lens to capture the cumulative nutritional impact of these interconnected determinants. The lack of a strong independent association between food security status (CARI) and individual anthropometric indicators may reflect limitations of cross-sectional measures in capturing chronic dietary inadequacy or seasonal variability [50,51,52]. In this context, the CIAF is particularly relevant for summarizing overall nutritional vulnerability in settings where economic hardship, mobility, and environmental stressors interact.

### 4.5. Implications for Policy and Programming

These findings have important implications for nutrition policy and programming in Djibouti. First, they support the routine integration of the CIAF into national nutrition surveillance systems alongside conventional anthropometric indicators, to better identify children experiencing multiple and overlapping forms of undernutrition [53,54]. Second, the marked disparities observed by region and household type highlight the need for geographically targeted and context-specific interventions, particularly among nomadic populations and in high-risk regions such as Obock. Finally, the strong age gradient underscores the urgency of strengthening interventions during early childhood, including maternal nutrition, optimal infant and young child feeding practices, and infection prevention [55]. Collectively, the CIAF provides a valuable tool to inform more holistic, efficient, and equitable nutrition strategies in Djibouti.

### 4.6. Strengths and Limitations

A major strength of this study is the use of nationally representative data combined with standardized anthropometric measurements, which enables robust estimation of undernutrition patterns across regions and population subgroups. The use of the CIAF allows a more comprehensive assessment of children’s nutritional vulnerability by capturing the coexistence of multiple anthropometric deficits, beyond conventional indicators considered separately.

Several limitations should nevertheless be acknowledged. The cross-sectional design does not allow causal inference, and residual confounding cannot be fully excluded [20]. Some potentially important determinants of undernutrition, such as maternal nutritional status, child dietary diversity, and recent morbidity, were not available and could not be included in the analyses. Furthermore, food security was assessed at the household level, which may not accurately reflect individual child food consumption or intra-household allocation and may fail to capture seasonal variations in food availability.

Several limitations should nevertheless be acknowledged. The cross-sectional design does not allow causal inference, and residual confounding cannot be fully excluded [20]. Some potentially important determinants of undernutrition—such as maternal nutritional status, child dietary diversity, and recent morbidity—were not available and could not be included in the analyses. In addition, micronutrient malnutrition could not be assessed because the 2023 survey database did not include biochemical or clinical indicators of micronutrient status. Furthermore, food security was assessed at the household level, which may not accurately reflect individual child food consumption, intra-household allocation, or seasonal variations in food availability. Finally, overweight and obesity were not included in the CIAF, as the index was applied in its original formulation focusing on anthropometric failure related to growth deficits, and no standardized definition of an extended CIAF including overnutrition has yet been universally adopted, which supports comparability with existing literature.

## 5. Conclusions

This study demonstrates that child undernutrition remains a major public health challenge in Djibouti, affecting more than one-third of children under five years of age when assessed using the CIAF.

The CIAF revealed a substantial burden of overlapping and multiple anthropometric deficits that would be underestimated if conventional indicators were considered in isolation. Undernutrition was strongly patterned by age, gender, household mobility, and geographic location, with particularly high vulnerability among children aged 6–47 months, those living in nomadic households, and those residing in regions such as Obock. These findings underscore the need for integrated, context-sensitive nutrition strategies that address both acute and chronic forms of malnutrition and explicitly target high-risk populations.

Incorporating the CIAF into routine nutrition surveillance and policy planning could substantially improve the identification of vulnerable children and the design of more effective, equitable interventions. Strengthening early-life nutrition, improving access to services for mobile populations, and addressing regional inequalities should be central priorities for accelerating progress toward national and global nutrition targets in Djibouti.

## Figures and Tables

**Table 1:** Category anthropometric failure in under five year using Composite Index of Anthropometric Failure (CIAF) [30].

Group	CIAD-Categories	Description of the Level
A	No failure	Normal WHZ, WAZ and HAZ
B	Stunting only	HAZ <−2 SD, but normal WAZ and WHZ
C	Wasting only	WHZ <−2 SD, but normal WAZ and HAZ
D	Underweight only	WAZ <−2 SD, but normal WHZ and HAZ
E	Stunting and underweight	HAZ and WAZ <−2 SD, but normal WHZ
F	Wasting and underweight	WHZ and WAZ <−2 SD, but normal HAZ
Y	Wasting, underweight and stunting	WHZ, WAZ and HAZ <−2 SD

**Table 2:** Prevalence of undernutrition according to conventional indices and CIAF.

	Both Genders (*n* = 2067) ^1^ % ^2^ (95% CI) ^3^	Girls (*n* = 1004) ^1^ % ^2^ (95% CI) ^3^	Boys (*n* = 1063) ^1^ % ^2^ (95% CI) ^3^	*p*-Value Girls vs. Boys ^4^
Wasting	9.8 (8.5–11.1)	8.0 (6.3–9.7)	11.6 (9.6–13.5)	0.006
Stunting	29.2 (27.3–31.2)	27.8 (25.0–30.6)	30.6 (27.8–33.3)	0.16
Underweight	19.9 (18.2–21.7)	19.0 (16.6–215)	20.8 (18.3–23.2)	0.31
A: No failure	63.2 (61.1–65.2)	66.0 (63–68.9)	60.5 (57.6–63.4)	0.06
B: Stunting only	13.9 (12.3–15.3)	13.0 (10.9–15)	14.6 (12.5–16.7)	
C: Wasting only	3.0 (2.3–3.8)	2.2 (1.3–3.2)	3.8 (2.7–4.9)	
D: Underweight only	1.1 (0.7–1.6)	1.4 (0.7–2.1)	0.9 (0.4–1.5)	
E: Stunting and underweight	12.0 (10.6–13.4)	11.7 (9.7–13.7)	12.3 (10.4–14.3)	
F: Wasting and underweight	3.4 (2.6–4.2)	2.7 (1.7–3.7)	4.1 (2.9–5.3)	
Y: Wasting, underweight and stunting	3.4 (2.6–4.2)	3.0 (2–4.1)	3.8 (2.7–4.9)	
Any anthropometric failure	36.8 (34.7–38.9)	34.2 (31.2–37.1)	39.3 (36.4–42.3)	0.02

^1^ Sample size. ^2^. Prevalence proportion (weighted estimates). ^3^. 95% confidence interval taking into account sampling design. ^4^ *p*-value comparing girls vs. boys.

**Table 3:** Crude and adjusted analysis of economic and sociodemographic factors associated with wasting among children under five years in Djibouti.

Variable	*n* ^1^	Prevalence	Crude Analysis				Adjusted Analysis		
		% ^2^	RRR ^3^	95% CI ^4^	*p*-Value ^5^	Overall *p*-Value ^6^	RRR ^3^	95% CI ^4^	*p*-Value ^5^
**Gender**									
Boys	1063	11.6	1			0.006	1		
Girls	1004	8.0	0.7	0.49–0.88	0.006		0.6	0.44–0.82	0.001
**Age (months)**									
6–23	664	13.3	0.9	0.56–1.30	0.47	0.001	0.8	0.54–1.28	0.42
24–47	1028	7.9	1.5	1.00–2.32	0.04		1.5	1.01–2.38	0.044
48–59	375	9.1	1				1		
**Area of residence**									
Rural	995	11.2	1			0.05	1		
Urban	1072	8.6	0.7	0.55–1.00	0.05		1.3	0.86–2.12	0.19
**Household type**									
Sedentary	1107	7.9	0.6	0.46–0.83	0.001	0.001	0.3	0.20–0.58	<0.0001
nomadic	960	12.1	1				1		
**Region**									
Dikhil	241	7.9	1			0.03	1		
Djibouti-city	147	5.4	1.5	0.80–2.70	0.20		1.6	0.87–3.10	0.12
Balbala	358	9.5	1.0	0.56–1.93	0.88		1.1	0.59–2.15	0.72
Ali-Sabieh	367	11.2	1.2	0.68–2.20	0.49		2.2	1.11–4.49	0.02
Tadjourah	500	10.0	0.7	0.28–1.57	0.36		1.0	0.42–2.79	0.85
Obock	226	15.5	2.1	1.18–3.86	0.01		3.1	1.64–5.98	0.0005
Arta	328	8.2	1.3	0.74–2.25	0.35		1.2	0.71–2.28	0.40
**Education level of household head**									
Low	1199	10.9	1			0.12	1		
Middle	239	7.5	0.7	0.39–1.11	0.11		0.4	0.17–1.42	0.19
High	629	8.6	0.8	0.54–1.06	0.11		0.5	0.16–1.95	0.37
**Occupation of household head**									
Large-scale trader	178	10.7	1			0.21	1		
Worker and employee	482	8.9	0.8	0.46–1.44	0.49		0.8	0.47–1.55	0.61
Senior middle manager	805	8.4	0.8	0.45–1.32	0.34		1.5	0.42–5.61	0.50
Unemployed/dependent aid	251	12.4	1.2	0.64–2.16	0.59		1.3	0.68–2.46	0.42
Small-scale trader	351	12.0	1.1	0.64–2.02	0.66		1.0	0.56–1.85	0.94
**Household wealth index**									
Low	688	10.6	1.3	0.90–1.86	0.15	0.28	0.7	0.41–1.18	0.18
Middle	685	10.5	1.3	0.89–1.85	0.17		1.0	0.67–1.55	0.91
High	694	8.4	1				1		
**Household income index**									
Low	555	11.4	1.5	1.03–2.22	0.03	0.06	1.3	0.85–2.21	0.18
Middle	804	10.6	1.4	0.98–2.00	0.06		1.3	0.86–2.01	0.19
High	708	7.8	1				1		
**Food security index CARI**									
Food secure	152	8.6	0.4	0.11–1.35	0.14	0.42	0.5	0.14–1.93	0.33
Marginally food secure	1415	10.1	0.5	0.15–1.43	0.18		0.7	0.22–2.30	0.58
Moderately food insecure	479	9.0	0.4	0.13–1.30	0.13		0.5	0.16–1.72	0.29
Severely food insecure	21	19.0	1				1		

^1^ Sample size. ^2^ Prevalence proportion (weighted estimates). ^3^ RRR. relative risk ratio (vs. reference category for which RRR = 1), taking the sampling design into account. ^4^ 95% Confidence Interval. ^5^ Crude or adjusted *p*-value for multinomial regression models. ^6^ Overall *p*-value for crude analyses.

**Table 4:** Crude and adjusted analysis of economic and sociodemographic factors associated with stunting among children under five years in Djibouti.

Variable	*n* ^1^	Prevalence	Crude Analysis				Adjusted Analysis		
		% ^2^	RRR ^3^	95% CI ^4^	*p*-Value ^5^	Overall *p*-Value ^6^	RRR ^3^	95% CI ^4^	*p*-Value ^5^
**Gender**									
Boys	1063	30.6	1				1		
Girls	1004	27.8	0.9	0.72–1.05	0.16	0.16	0.8	0.67–1.00	0.056
**Age (months)**								-	
6–23	664	27.7	1.7	1.27–2.21	0.0001		1.7	1.26–2.22	0.0001
24–47	1028	32.7	1.3	0.98–1.78	0.06	0.0005	1.4	1.00–1.85	0.04
48–59	375	22.4	1				1		
**Area of residence**									
Rural	995	34.1	1				1		
Urban	1072	24.7	0.6	0.52–0.76	<0.0001	<0.0001	1.0	0.72–1.30	0.86
**Household type**		24.4							
Sedentary	1107	34.8	0.6	0.50–0.73	<0.0001	<0.0001	0.7	0.47–0.93	0.01
nomadic	960	24.4	1				1		
**Region**									
Dikhil	241	20.4	1			0.0004	1		
Djibouti-city	147	26.3	0.7	0.46–1.03	0.07		0.8	0.50–1.15	0.20
Balbala	358	23.2	0.9	0.60–1.25	0.45		0.9	0.64–1.38	0.76
Ali-Sabieh	267	30.3	0.8	0.57–1.17	0.28		1.2	0.76–1.80	0.45
Tadjourah	500	34.6	0.6	0.36–0.96	0.03		0.9	0.51–1.52	0.67
Obock	226	36.3	1.3	0.89–1.92	0.17		1.6	1.03–2.41	0.03
Arta	328	27.4	1.2	0.87–1.69	0.24		1.2	0.86–1.73	0.25
**Education level of household head**									
Low	1199	31.3	1				1		
Middle	239	28.0	0.9	0.62–1.16	0.32	0.04	1.3	0.71–2.19	0.42
High	629	25.8	0.8	0.61–0.94	0.01		1.3	0.65–2.55	0.45
**Occupation of household head**									
Large-scale trader	178	30.3	1			0.03	1.1	0.71–1.58	0.74
Worker and employee	482	25.6	1.1	0.72–1.53	0.78		0.8	0.36–1.52	0.44
Senior middle manager	805	34.7	0.8	0.58–1.19	0.32		1	0.66–1.59	0.89
Unemployed/dependent aid	251	29.2	1.3	0.84–1.94	0.23		1	0.67–1.53	0.92
Small-scale trader	351	32.2	1.2	0.77–1.70	0.48		1.3	0.71–2.19	0.42
**Household wealth index**									
Low	688	35.5	1.9	1.48–2.38	<0.0001	<0.0001	1.3	0.92–1.80	0.13
Middle	685	29.6	1.4	1.13–1.83	0.003		1.3	0.97–1.68	0.08
High	694	22.6	1				1		
**Household income index**									
Low	555	34.1	1.6	1.24–2.02	0.0001	<0.0001	1.1	0.81–1.52	0.48
Middle	804	30.0	1.3	1.04–1.65	0.01		1	0.78–1.33	0.87
High	708	24.6	1				1		
**Food security index CARI**									
Food secure	152	28.9	0.7	0.25–1.70	0.39	0.41	1	0.39–2.76	0.93
Marginally food secure	1415	28.3	0.6	0.26–1.55	0.32		0.9	0.36–2.27	0.84
Moderately food insecure	479	31.7	0.8	0.30–1.86	0.54		0.9	0.36–2.33	0.87
Severely food insecure	21	38.1	1				1		

^1^ Sample size. ^2^ Prevalence proportion (weighted estimates). ^3^ RRR. relative risk ratio (vs. reference category for which RRR = 1), taking the sampling design into account. ^4^ 95% Confidence Interval. ^5^ Crude or adjusted *p*-value for multinomial regression models. ^6^ Overall *p*-value for crude analyses.

**Table 5:** Crude and adjusted analysis of economic and sociodemographic factors associated with underweight among children under five years in Djibouti.

Variable	*n* ^1^	Prevalence	Crude Analysis				Adjusted Analysis		
		% ^2^	RRR ^3^	95% CI ^4^	*p*-Value ^5^	Overall *p*-Value ^6^	RRR ^3^	95% CI ^4^	*p*-Value ^5^
**Gender**									
Boys	1063	20.8	1			0.31	1		
Girls	1004	19.0	0.9	0.72–1.11	0.31		0.8	0.65–1.02	0.08
**Age (months)**									
6–23	664	19.6	1.2	0.85–1.56	0.33	0.61	1.2	0.84–1.57	0.35
24–47	1028	20.7	1.1	0.78–1.49	0.64		1.1	0.79–1.54	0.54
48–59	375	18.4	1				1		
**Area of residence**									
Rural	995	24.0	1			<0.0001	1		
Urban	1072	16.1	0.6	0.48–0.75	<0.0001		1.0	0.72–1.38	0.98
**Household type**									
Sedentary	1107	15.9	0.6	0.46–0.72	<0.0001	<0.0001	0.7	0.45–0.96	0.03
nomadic	960	24.6	1				1		
**Region**									
Dikhil	241	6.8	1			<0.0001	1		
Djibouti-city	147	16.8	0.8	0.49–1.17	0.21		0.7	0.45–0.96	0.03
Balbala	358	18.4	0.7	0.49–1.12	0.15		0.9	0.54–1.35	0.51
Ali-Sabieh	267	22.8	0.7	0.45–1.02	0.06		0.8	0.51–1.23	0.31
Tadjourah	500	22.2	0.2	0.12–0.50	0.0001		1.0	0.58–1.53	0.83
Obock	226	30.1	1.5	0.96–2.20	0.07		0.4	0.16–0.75	0.007
Arta	328	18.0	1.0	0.66–1.39	0.84		1.7	1.07–2.65	0.02
**Education level of household head**									
Low	1199	22.0	1			0.02	1		
Middle	239	16.7	0.7	0.49–1.02	0.06		0.8	0.40–1.57	0.51
High	629	17.2	0.7	0.57–0.94	0.01		0.8	0.37–1.92	0.69
**Occupation of household head**									
Large-scale trader	178	19.3	1			0.01	1		
Worker and employee	482	16.9	0.9	0.55–1.29	0.45		0.9	0.59–1.41	0.68
Senior middle manager	805	26.3	0.7	0.48–1.08	0.11		1.1	0.44–2.51	0.90
Unemployed/dependent aid	251	21.9	1.3	0.80–2.00	0.29		1.2	0.73–1.90	0.49
Small-scale trader	351	22.2	1.0	0.65–1.57	0.93		1.0	0.62–1.54	0.95
**Household wealth index**									
Low	688	25.0	1.9	1.47–2.53	<0.0001	<0.0001	1.2	0.80–1.72	0.40
Middle	685	20.1	1.5	1.10–1.93	0.008		1.3	0.91–1.73	0.16
High	694	14.7	1				1		
**Household income index**									
Low	555	24.5	1.7	1.26–2.21	0.0003	0.001	1.2	0.87–1.77	0.22
Middle	804	20.0	1.3	0.99–1.68	0.05		1.1	0.77–1.44	0.72
High	708	16.2	1				1		
**Food security index CARI**									
Food secure	152	16.4	0.8	0.26–2.69	0.76	0.63	1.2	0.36–3.94	0.77
Marginally food secure	1415	19.9	1.1	0.35–3.15	0.92		1.5	0.48–4.53	0.48
Moderately food insecure	479	21.3	1.2	0.37–3.49	0.80		1.3	0.42–4.06	0.63
Severely food insecure	21	19.0	1				1		

^1^ Sample size. ^2^ Prevalence proportion (weighted estimates). ^3^ RRR. relative risk ratio (vs. reference category for which RRR = 1), taking the sampling design into account. ^4^ 95% Confidence Interval. ^5^ Crude or adjusted *p*-value for multinomial regression models. ^6^ Overall *p*-value for crude analyses.

**Table 6:** Crude and adjusted analysis of economic and sociodemographic factors associated with any failure among children under five years in Djibouti.

Variable	*n* ^1^	Prevalence	Crude Analysis				Adjusted Analysis		
		% ^2^	RRR ^3^	95% CI ^4^	*p*-Value ^5^	Overall *p*-Value ^6^	RRR ^3^	95% CI ^4^	*p*-Value ^5^
**Gender**									
Boys	1063	20.8	1			0.015	1		
Girls	1004	19.0	0.8	0.66–0.95	0.015		0.7	0.61–0.89	0.001
**Age (months)**									
6–23	664	19.6	1.4	1.10–1.82	0.007	0.02	1.4	1.08–1.82	0.009
24–47	1028	20.7	1.4	1.04–1.78	0.02		1.4	1.06–1.85	0.01
48–59	375	18.4	1				1		
**Area of residence**									
Rural	995	24.0	1			<0.0001	1		
Urban	1072	16.1	0.6	0.51–0.73	<0.0001		1.0	0.72–1.2	0.75
**Household type**									
Sedentary	1107	15.9	1.7	1.41–2.02	<0.0001	<0.0001	0.6	0.45–0.85	0.003
nomadic	960	24.6	1				1		
**Region**									
Dikhil	7.9	6.8	1			<0.0001	1		
Djibouti-city	5.4	16.8	0.7	0.48–1.08	0.04		0.7	0.50–1.09	0.13
Balbala	9.5	18.4	0.8	0.54–1.08	0.12		0.8	0.55–1.15	0.23
Ali-Sabieh	11.2	22.8	0.8	0.55–1.08	0.14		1.1	0.75–1.6	0.55
Tadjourah	10.0	22.2	0.5	0.34–0.84	0.006		0.8	0.48–1.32	0.38
Obock	15.5	30.1	1.3	0.92–1.92	0.12		1.6	1.08–2.42	0.01
Arta	8.2	18.0	1.1	0.82–1.53	0.47		1.1	0.77–1.49	0.68
**Education level of household head**									
Low	1199	22.0	1			0.01	1		
Middle	239	16.7	0.8	0.59–1.07	0.13		1.1	0.64–1.92	0.69
High	629	17.2	0.7	0.61–0.91	0.005		1.2	0.63–2.32	0.56
**Occupation of household head**									
Large-scale trader	178	19.3	1			0.006	1		
Worker and employee	482	16.9	1.0	0.67–1.36	0.80		0.9	0.68–1.43	0.96
Senior middle manager	805	26.3	0.8	0.55–1.08	0.13		0.7	0.38–1.55	0.47
Unemployed/dependent aid	251	21.9	1.3	0.85–1.86	0.24		1.0	0.70–1.61	0.74
Small-scale trader	351	22.2	1.1	0.76–1.61	0.57		1.0	0.68–1.47	0.99
**Household wealth index**									
Low	688	25.0	1.9	1.48–2.31	<0.0001	<0.0001	1.2	0.87–1.63	0.27
Middle	685	20.1	1.4	1.15–1.80	0.001		1.3	0.97–1.62	0.08
High	694	14.7	1				1		
**Household income index**									
Low	555	24.5	1.6	1.28–2.03	<0.0001	0.0002	1.1	0.85–1.42	0.44
Middle	804	20.0	1.4	1.10–1.69	0.003		1.2	0.87–1.63	0.27
High	708	16.2	1				1		
**Food security index CARI**									
Food secure	152	16.4	0.6	0.23–1.47	0.25	0.57	0.9	0.35–2.39	0.87
Marginally food secure	1415	19.9	0.6	0.26–1.48	0.29		0.9	0.38–2.25	0.86
Moderately food insecure	479	21.3	0.7	0.28–1.64	0.39		0.9	0.34–2.09	0.72
Severely food insecure	21	19.0	1				1		

^1^ Sample size. ^2^ Prevalence proportion (weighted estimates). ^3^ RRR. relative risk ratio (vs. reference category for which RRR = 1), taking the sampling design into account. ^4^ 95% Confidence Interval. ^5^ Crude or adjusted *p*-value for multinomial regression models. ^6^ Overall *p*-value for crude analyses.

**Table 7:** Summary of key factors associated with child undernutrition across anthropometric indicators (adjusted analysis).

Predictor	Wasting	Stunting	Underweight	CIAF
Gender (boys vs. girls)	↑	—	—	↑
Age (6–23 months vs. 48–59 months as reference)	—	↑	—	↑
Age (24–47 months vs. 48–59 months as reference)	↑	↑	—	↑
Area of residence (rural vs. urban)	±	±	±	±
Household type (nomadic vs. sedentary)	↑	↑	↑	↑
Region (Djibouti city vs. Dikhil as reference)	—	—	↑	±
Region (Ali Sabieh vs. Dikhil as reference)	↑	—	—	—
Region (Obock vs. Dikhil as reference)	↑	↑	↑	↑
Region (Arta vs. Dikhil as reference)	—	—	↑	—
Region (other regions vs. Dikhil as reference)	—	—	—	±
Education level of household head (lowest vs. highest)	—	±	±	±
Occupation of household head (large scale traders vs. others)	—	—	—	—
Household wealth index (highest vs. lowest)	—	±	±	±
Household income index (highest vs. lowest)	—	±	±	±
Food security status (CARI) (severely food insecure vs. others)	—	—	—	—

Values indicate direction of statistically significant associations in the final multivariate logistic regression models. ↑: higher odds of malnutrition outcome (*p* < 0.05); —: not statistically significant in adjusted models; ±: significance observed in some categories or attenuated after adjustment. Models were adjusted for child age and genre, household characteristics, socioeconomic factors, region, and food security status as described in the Methods section. CIAF: Composite Index of Anthropometric Failure.

## Data Availability

The original contributions presented in this study are included in the article. Further inquiries can be directed to the corresponding author.

## Abbreviations

The following abbreviations are used in this manuscript:

CARI	Consolidated Approach to Reporting Indicators
CIAF	Composite Index of Anthropometric Failure
EA	Enumeration Areas
GDP	Gross Domestic Product
NFHS-5	National Family Health Survey-5, 2019-21
PCA	Principal Component Analysis
VIFs	Variance Inflation Factors
WASH	Water, sanitation and hygiene
